# H4K12 Lactylation Activated‐*Spp1* in Reprogrammed Microglia Improves Functional Recovery After Spinal Cord Injury

**DOI:** 10.1111/cns.70232

**Published:** 2025-02-12

**Authors:** Xiaokun Wang, Geliang Zhou, Junjun Xiong, Wu Ye, Yu Gao, Haofan Wang, Dishui Pan, Yongjun Luo, Zheng Zhou

**Affiliations:** ^1^ Department of Orthopedics The First Affiliated Hospital of Nanjing Medical University Nanjing Jiangsu China; ^2^ Department of First Clinical Medical College of Nanjing Medical University Nanjing Jiangsu China; ^3^ Department of Orthopedics The Fourth Affiliated Hospital of Soochow University Suzhou Jiangsu China; ^4^ Emergency and Critical Care Medicine Department The First Affiliated Hospital of Nanjing Medical University Nanjing Jiangsu China

**Keywords:** histone lactylation, metabolic reprogramming, microglia, spinal cord injury, SPP1

## Abstract

**Background:**

Spinal cord injury (SCI) is a severe condition leading to significant disability and high mortality. The role of the secreted phosphoprotein 1 (SPP1) signaling pathway in SCI, which is quickly activated after injury, is critical for intercellular communication but remains poorly understood.

**Aims:**

This study aimed to explore the function and regulatory mechanisms of the SPP1 signaling pathway in SCI and investigate its potential as a therapeutic target for improving functional recovery after injury.

**Materials and Methods:**

Single‐cell RNA sequencing (scRNA‐seq) was employed to identify ligands and receptors of the SPP1 signaling pathway, particularly in microglia/macrophages. Recombinant SPP1 (rSPP1) was used in vitro and in vivo to assess its effects on neuronal maturation, mitochondrial energy in axons, and functional recovery after SCI. Pseudotime analysis was conducted to examine the role of *Spp1* in microglial activation and proliferation. DNA‐pulldown and in vitro experiments were performed to investigate the upstream regulatory proteins of *Spp1*.

**Results:**

The SPP1 signaling pathway is primarily localized in microglia after SCI, with rSPP1 promoting neuronal maturation and enhancing mitochondrial function in axons. Injection of rSPP1 into the injured spinal cord resulted in significant improvement in functional recovery. Pseudotime analysis indicated that *Spp1* is involved in the activation and proliferation of microglia. Histone H4 lysine 12 lactylation (H4K12la) was found to promote the transcription of *Spp1* in reprogrammed microglia postinjury.

**Discussion:**

Our findings reveal a novel regulatory mechanism involving *Spp1* in SCI, particularly its role in microglial activation, mitochondrial function, and glycolytic reprogramming. This new insight provides a deeper understanding of its contribution to the injury response.

**Conclusion:**

This study uncovers a previously unreported mechanism of *Spp1* in SCI, offering a potential therapeutic target for SCI.

## Graphical abstract

Following spinal cord injury, microglia shift into the “activated” and “proliferative” state. Glycolytic reprogramming triggers H4K12 lactylation, which upregulates the transcription of *Spp1*. SPP1 protein is subsequently released to facilitate intercellular communication.

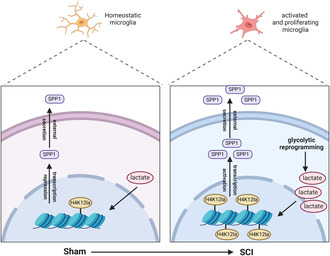

## 1 Introduction

Spinal cord injury (SCI) is a devastating trauma with severe repercussions, causing lasting impairments in sensory and motor capabilities [1, 2]. SCI is generally considered irreversible after onset, resulting in the partial or complete loss of movement, sensation, and bowel and urinary functions below the injury site, posing significant challenges for individuals and society [3]. Hence, there is an urgent need for a deeper insight into the molecular mechanisms following SCI.

SCI is a multi‐temporal, multi‐component and complicated pathophysiological event. Various biological signals and cellular interactions become abnormally active after SCI, playing distinct roles over time [4]. Over the past decade, bulk RNA sequencing (bulk‐RNA seq) of spinal cord tissues at different time points after SCI has revealed various molecular and cytopathological events, helping to elucidate the pathophysiological mechanisms of the SCI microenvironment. Recently, advances in single‐cell RNA sequencing (scRNA‐seq) technologies have allowed exploration of cellular diversity in the spinal cord, expanding our understanding of SCI [5]. Unlike bulk‐RNA seq, scRNA‐seq analysis precisely reveals specific changes in various cell types and shows cellular events in SCI tissues at single‐cell resolution across different time points post‐injury. Thus, the intercellular communications and specific functions of receptor‐ligand signaling pathways could be identified.

Secreted phosphoprotein 1 (SPP1), also known as osteopontin (OPN), is an acidic phosphoprotein widely expressed in various cells and tissues. SPP1 is involved in processes such as inflammation, wound healing, and tumorigenesis, which are essential for tissue homeostasis and repair [6]. Despite the incomplete understanding of its precise function, amounts of researches have unveiled the significance of SPP1 in the regeneration of central nervous system (CNS). Schroeter et al. reported that SPP1 plays a neuroprotective role in ischemic stroke, and increased thalamic neurodegeneration was observed in *Spp1*‐deficient mice [7]. Additionally, SPP1 has shown strong pro‐regeneration potential in both optic [8] and sciatic nerve injuries [9]. The upregulation of *Spp1* post‐SCI is well established [10, 11, 12, 13]. Indeed, *Spp1* plays a crucial role in post‐SCI recovery through various mechanisms. It enhances angiogenesis through VEGF and AKT pathways and shows potential in mitigating neuropathic pain after SCI [14]. The co‐overexpression of *SPP1*, *IGF1*, and *CNTF* boosts the impact of dental pulp stem cells (DPSC) in SCI [15]. Although extensive studies have demonstrated the potential neuroprotective benefits in SCI, the underlying mechanisms and its effects on other functional aspects remain unclear.

The enhancement of glycolysis is a key metabolic adaptation in microglia after SCI, promoting both morphological and functional recovery [16]. Lactate, a key byproduct of glycolytic reprogramming, has been identified as the substrate for histone lactylation [17]. Recently, histone lactylation has been implicated in the regulation of diverse biological processes including CNS function [18], tumorigenesis [19], and somatic cell reprogramming [20]. Interestingly, histone H4 lysine‐12 lactylation (H4K12la) was proved to exacerbate glycolytic dysregulation in microglia [21].

In this study, we revealed the activation of the SPP1 pathway in intercellular communication with scRNA‐seq analysis and demonstrated that SPP1 protein levels increased following SCI, promoting mitochondrial energetics, enhancing neuronal maturation, preventing inflammatory cell infiltration, and improving functional recovery, as shown by both in vitro and in vivo experiments. Mechanistically, H4K12la promoted the transcription of *Spp1* in microglia, leading to increased SPP1 secretion into the extracellular matrix and enhanced cellular communication. In summary, our study uncovers the underlying mechanism of *Spp1* activation, which might provide promising strategies for SCI treatment.

## 2 Materials and Methods

### 2.1 Microarray Data

Open‐access sequencing data were collected from the Gene Expression Omnibus (GEO) database and figshare database. Three scRNA‐seq datasets (GSE162610, GSE189070, and figshare 17,702,045) and three bulk‐RNA seq datasets (GSE5296, GSE42828, and GSE47681) were retrieved according to the following inclusions: (1) mice were treated with thoracic SCI, and without drug or other intervention. (2) scRNA‐seq or bulk‐RNA seq was performed in the subacute phase (within 14 days). (3) Sham‐injury groups were conducted for a comparison with SCI.

### 2.2 scRNA‐Seq Data Processing

scRNA‐seq data was processed with “Seurat” package. The quality control of single cell samples was conducted based on the following criteria: (1) each cell expresses more than 200 genes; (2) the same gene is expressed in a minimum of three cells. (3) percentage of hemoglobin genes < 0.1; (4) percentage of mitochondrial genes < 0.1. Normalization and standardization were applied to the filtered gene expression matrix and 2000 highly variable genes were identified for subsequent analysis. Principal components analysis (PCA) was performed, and then the initial 10 principal components were applied for UMAP visualization. Shared nearest neighbor (SNN) clustering was performed with a resolution of 0.8, followed by manual clustering based on canonical gene markers annotation. Cellular communication profiling was calculated with “CellChat” package. By deducing and visualizing the differences in receptor‐ligand signaling pathways across fundamental cell subsets, and ascertaining the specific functions of these pathways in distinct subgroups (sham and SCI), we unraveled the communication patterns amidst diverse cell clusters.

### 2.3 Pseudotime Trajectory Analysis

The R package “monocle2” was employed to depict single‐cell differentiation trajectories through pseudotemporal ordination, with each trajectory marked by a specific starting and ending point. Cells were hierarchically clustered with subsequent dimensionality reduction conducted by “DDRTree” method. Ultimately, a reductive view of the differentiation trajectories was obtained. Additionally, we scrutinized the expression fluctuations of *Spp1* in different cell differentiation processes simultaneously.

### 2.4 SCI Model and Treatment

SCI model was performed as previously described [22]. Anesthesia was induced using isoflurane inhalation prior to performing a laminectomy at the T8 level to expose the spinal cord. The SCI model was created by dropping a 5 g rod onto the spinal cord from a height of 6.5 cm using a spinal cord percussion device. The mice bladders were auxiliary emptied twice a day until normal urination resumed.

### 2.5 Functional Behavioral Analysis

The mice were housed under a 12‐h light–dark cycle with ad libitum access to food and water. Prior to behavioral tests, the mice were acclimated to the testing room for 1 h. Blind scoring was employed to prevent observers from knowing the treatment assignments of the mice.

The Basso Mouse Scale (BMS) score was assessed by two blinded evaluators at 1, 3, 7, 14, and 28 days post‐injury. Scores varied from 0 for complete paraplegia to 9 for normal function.

Following SCI, the rotarod test was conducted to evaluate balance and motor coordination. The mice were placed on a speed‐adjustable rotarod (0–40 rpm), and the speed of the rotarod and the endurance time of the mice were recorded. Prior to the two test trials, each mouse underwent a single practice trial, with a 20‐min interval between trials. The final score for each mouse was calculated by averaging the results of the two individual tests.

Footprint analysis was performed as previously described [22]. Blue dye was applied to the forelimbs, while red dye was used for the hindlimbs. The mice were then allowed to walk straight on a sheet of white paper. Stride lengths and widths were measured and analyzed separately.

The motor evoked potentials (MEPs) were assessed with electromyography testing 28 days post‐SCI. At the rostral ends of spinal cord, flexor of biceps femoris, distal tendon of hindlimb muscle, and under skin, the stimulation, recording, reference, and grounding electrode were placed, respectively. MEPs were induced by a single stimulation (0.5 mA, 0.5 ms, 1 Hz), with subsequent measurement of amplitude and latency to evaluate the functional recovery.

### 2.6 Western Blot Analysis

The concentration of total protein extracted from cells or spinal cord tissues was measured with the BCA method (Thermo Fisher Scientific). Equal protein was separated with SDS‐PAGE, and then transferred to PVDF membranes (EMD Millipore Corp). PVDF membranes were incubated with specific primary antibodies at 4°C overnight after 5% BSA blocking. Incubation with corresponding secondary antibodies (1:10000; Jackson ImmunoResearch) for 2 h was next, accompanied by treatment with enhanced chemiluminescent reagent (Millipore).

### 2.7 Immunofluorescence Staining

The hearts of SCI mice were perfused with 0.9% saline, followed by 4% paraformaldehyde. Subsequently, the injured spinal cords were removed and immersed in 4% paraformaldehyde overnight. The spinal cords were dehydrated sequentially in 15% and 30% sucrose solutions, embedded in OCT compound, and dissected into 14 μm thick sections. Spinal sections were blocked with 5% BSA plus 0.3% Triton X‐100 initially before being incubated with primary antibodies (anti‐IBA1, anti‐GFAP, anti‐CD68, anti‐NF, and anti‐H4K12la) overnight at 4°C. Conjugated secondary antibodies (AlexaFluor 488 or AlexaFluor 594; 1:300; Jackson ImmunoResearch) were applied, and DAPI (1:1000; Invitrogen) was utilized for counterstaining. Images were captured with Thunder Imager (THUNDER DMi8, LEICA).

### 2.8 RNAscope In Situ Hybridization

Fluorescent in situ hybridization was performed with RNAscope kit (ACD Biosystems) according to the manufacturer's protocol. Mice spinal cord slices were exposed to hydrogen peroxide for 10 min at room temperature, followed by target retrieval at 100°C for 15 min and treatment with protease IV solution at 40°C for 30 min. Subsequently, the slices underwent hybridization with pre‐designed RNA probes (mouse *Spp1*), followed by signal amplification using TSA reagent and horseradish peroxidase. Immunofluorescence staining was performed after hybridization, then slices were incubated with IBA1 primary antibodies and conjugated secondary antibodies. DAPI (1:1000; Invitrogen) was employed for counterstaining, and image acquisition was performed with the Thunder Imager (THUNDER DMi8, LEICA).

### 2.9 Antibodies

The antibodies used for immunoblotting in this study were anti‐β‐actin (1:2000, Servicebi), anti‐SPP1 (1:500, Servicebio), anti‐Pan Kla (1:500, PTM BIO), anti‐H4K12la (1:2000, Servicebi), anti‐H4K8la (1:2000, Servicebio), anti‐H4K5la (1:2000, PTM BIO), and anti‐H4 (Abcam). Antibodies used for immunofluorescence staining were anti‐IBA1 (1:1000, Abcam), anti‐GFAP (1:1000, Cell Signaling Technology), anti‐CD68 (1:1000, Abcam), anti‐NF (1:1000, Abcam), and anti‐H4K12la (1:1000, PTM BIO).

### 2.10 Primary Neuron and Microglia Culture

Primary neurons were cultured in a manner consistent with previous descriptions [23]. Briefly, the brains of neonatal mice were promptly dissected, and cortices were then sectioned into 0.5–1 mm^3^ pieces. These sections were then digested with 2 mg/mL papain and 0.5 mg/mL Dnase for 20 min at 37°C in a shaking incubator. Centrifugation was performed at 1000 rpm for 5 min at 4°C to acquire cell suspensions, which was then filtered through a 70‐μm strainer. Then neurons were then seeded onto poly‐D‐lysine‐coated dishes and cultured with B‐27 Plus Neuronal Culture System at 37°C with 5% CO_2_. Half of the medium was replaced every 2 days.

For primary microglia culture, cells were prepared as above described [24], then cultured in T75 flasks at 37°C with 5% CO_2_. After 14 days of in vitro culture, mature microglia were harvested by shaking the flasks at 200 rpm for 2 h. The purified microglia were subsequently cultured in DMEM at 37°C with 5% CO_2_, with medium renewal every 2 days.

### 2.11 Isolation of Microglia From Mouse Spinal Cords

Mice were anesthetized and perfused with ice‐cold PBS to remove circulating blood cells. Spinal cords were dissected and digested in 10 mL of digest medium (HBSS with 1 mg/mL collagenase IV and 0.5 mg/mL DNase) for 20 min at 90 rpm. The resulting suspension was passed through 70 and 40‐μm strainers, and myelin debris was cleared via density centrifugation (1200 *g*, low acceleration/brake) using 30% and 70% Percoll for 30 min. Then cell pellet was washed in PBS. Microglia were subsequently labeled with anti‐CD11b‐coated MicroBeads (130‐093‐636, Miltenyi Biotec) on ice and separated using MACS according to the manufacturer's protocol.

### 2.12 Stimulation of Lipopolysaccharide (LPS) and IL‐4

Following overnight incubation in serum‐free conditions, primary microglial cultures were treated with either 100 ng/mL purified LPS, 20 ng/mL IL‐4, or PBS control for 4 h at 37°C and 5% CO_2_. Subsequently, the cultured cells were harvested for Western blot analysis.

### 2.13 Recombinant Protein Administration and Adeno‐Associated Virus (AAV) Infection

Mouse recombinant SPP1 (rSPP1, Abcam) or saline (vehicle) were intraspinally microinjected into the injury site. rSPP1 was injected into two sites (one on each side of the spinal cord), 0.6 mm below the surface, at a rate of 0.1 μL/min using glass micropipettes (with tips ground to 50–100 μm). The micropipettes were connected via high‐pressure tubing (Kopf) to 10 μL syringes, controlled by microinfusion pumps. Tract‐tracing of propriospinal neurons was performed by injection of AAV2/5 enhanced green fluorescent protein (EGFP) injected into the same rostral segments targeted with rSPP1 injections as described above.

### 2.14 Sholl Analysis

Fluorescent images depicting cultured neurons were acquired using the confocal microscope. The morphological reconstruction of Tuj1 positive neurons was carried out utilizing ImageJ software, as described previously [25]. In summary, by employing circles with diameters increasing in 20 μm increments from the center of the cell body, the number of intersections was measured.

### 2.15 Axon Microfluid

We cultured primary neurons on the soma side of the microfluidic devices (xonamicrofluidic) and subjected the axons in the axon chambers to axotomy through vacuum aspiration at 10 days to assess rSPP1 impact on axonal regeneration in vitro. The axon chambers were replenished with pre‐warmed culture media and treated for 24 h with or without rSPP1. The evaluation of axonal regrowth following axotomy in the terminal chambers was performed using Tuj1 staining.

### 2.16 Oxygen Glucose Deprivation and Reperfusion Model (OGD/R)

As described previously [26], OGD/R model was utilized to simulate spinal cord ischemia–reperfusion injury (SCIRI) in vitro. Briefly, primary neurons were cultured in a sugar‐free medium, transferred to a sealed chamber, and exposed to a continuous flow of mixed gas (95% N_2_/5% CO_2_) for 15 min. Subsequently, the medium was switched to normal medium, and the cells were relocated to a normal incubator. Neurons were cultured for 0–2 h for subsequent analysis.

### 2.17 Calcein‐AM/PI Double Staining

Cell death was visualized using propidium iodide (PI) (Beyotime), while viable cells were quantified with Calcein‐acetoxymethyl ester (Calcein‐AM) (Beyotime) and then examined through fluorescence microscopy.

### 2.18 GO‐Ateam2 ATP Signal Analysis and ATP Measurement

Primary neurons were transfected with an adenovirus that contained the pCMV‐Mito‐AT1.03 vector for real‐time monitoring of axonal ATP levels. Captured images were processed with ImageJ software to outline the axon region. The Image Calculator function divided the YFP signal (527 nm) by the CFP signal (475 nm) to create a representative image, which was then transformed into a heatmap using the “fire” lookup table.

### 2.19 Seahorse Extracellular Flux Analysis

The Seahorse FX24 Extracellular Flux Analyzer was utilized as previously described [22]. Oxygen consumption rate (OCR) traces was conducted by stepwise addition of oligomycin (an ATP synthase blocker), carbonyl cyanide 4‐(trifluoromethoxy) phenylhydrazone (FCCP, a mitochondrial uncoupler), antimycin A and rotenone (A&R, inhibitors of complex I and III). The basal respiration, ATP production, maximal respiratory, and spare capacity were determined with XFe Wave software (Seahorse Biosciences).

### 2.20 Single‐Gene GSEA Analysis Based on Hallmark

The Hallmark module in the Molecular Signatures Database (MsgDB) organizes gene sets into specific biological features and signaling pathways using known biological processes, signaling pathways, and disease characteristics as criteria [27]. Utilizing the aforementioned scRNA‐seq data, single‐gene GSEA analysis was performed to investigate *Spp1*‐related gene sets in microglia and macrophages. Briefly, the correlation coefficients between *Spp1* and all other genes in microglia or macrophages were calculated to generate the gene lists, and the single‐gene GSEA analysis was then performed based on 50 Hallmark pathways. Applying the criteria of an absolute *p* < 0.05, significant enrichment pathways were identified.

### 2.21 DNA Pull Down Assay

The DNA pull‐down assay utilized a DNA pull‐down test kit (Gzscbio). Initially, the *Spp1* promoter sequence was PCR amplified and biotin‐labeled. The biotin‐labeled *Spp1* promoter was then incubated with Dynabeads M‐280 Streptavidin beads from Thermo Fisher Scientific. The biotinylated DNA was subsequently incubated with protein lysates overnight at 4°C with gentle agitation. After washing and separation, the proteins bound to bead‐DNA complexes were analyzed using silver staining and mass spectrometry.

### 2.22 Measurement of Lactate Levels

Isolated microglia were homogenized with lysis buffer, sonicated at 300 W (3 s on and 7 s off) for 3 min on ice, and then centrifuged at 12,000 *g* for 10 min at 4°C. The collected supernatants were used to measure lactate levels utilizing a Lactate Colorimetric Assay Kit (K607‐100, Biovision).

### 2.23 qChIP

The ChIP assay was conducted using the ChIP assay kit (P2078, Beyotime) in accordance with the manufacturer's protocol. Briefly, microglia isolated from SCI and sham mice were crosslinked with 1% formaldehyde for 10 min at room temperature, and the crosslinking reaction was quenched by adding glycine to a final concentration of 125 mM for 5 min. The cells were then lysed with SDS lysis buffer containing 1 mM PMSF to obtain whole cell lysates. The lysates were subjected to sonication to shear the genomic DNA into chromatin fragments of 400–800 bp. Subsequently, cross‐links between proteins and DNA were reversed by heating, and the chromatin was extracted using a phenol‐chloroform method. The extracted chromatin was then diluted and incubated overnight at 4°C on a rotator with either IgG (as a control) or H4K12la antibodies. Following incubation, the chromatin‐antibody complexes were washed sequentially with low‐salt, high‐salt, lithium chloride immune complex washing buffers, and TE buffer. The chromatin complexes were then eluted, and the DNA was purified using a DNA purification kit (D0033, Beyotime). Finally, the purified DNA was quantified by real‐time PCR.

### 2.24 Statistical Analysis

The GraphPad software 9.0 (GraphPad Software Inc.) was applied for statistical analysis. We evaluated the distribution of all datasets for normality as a prerequisite to analysis. A single‐sample Student's *t*‐test (for normally distributed data) or a single‐sample Wilcoxon signed‐rank test (for non‐normally distributed data) was employed to compare the average signaling pathway activation values after SCI to the uninjured group. A two‐tailed unpaired Student's *t*‐test was conducted for comparisons between two groups, while one‐way or two‐way analysis of variance (ANOVA) with post hoc Bonferroni correction was employed for multivariate analysis. Results were presented as mean ± standard error of the mean (SEM), and significance was determined by *p* < 0.05.

## 3 Results

### 3.1 SCI Promotes SPP1 Signaling Pathway Activation

**FIGURE 1:**
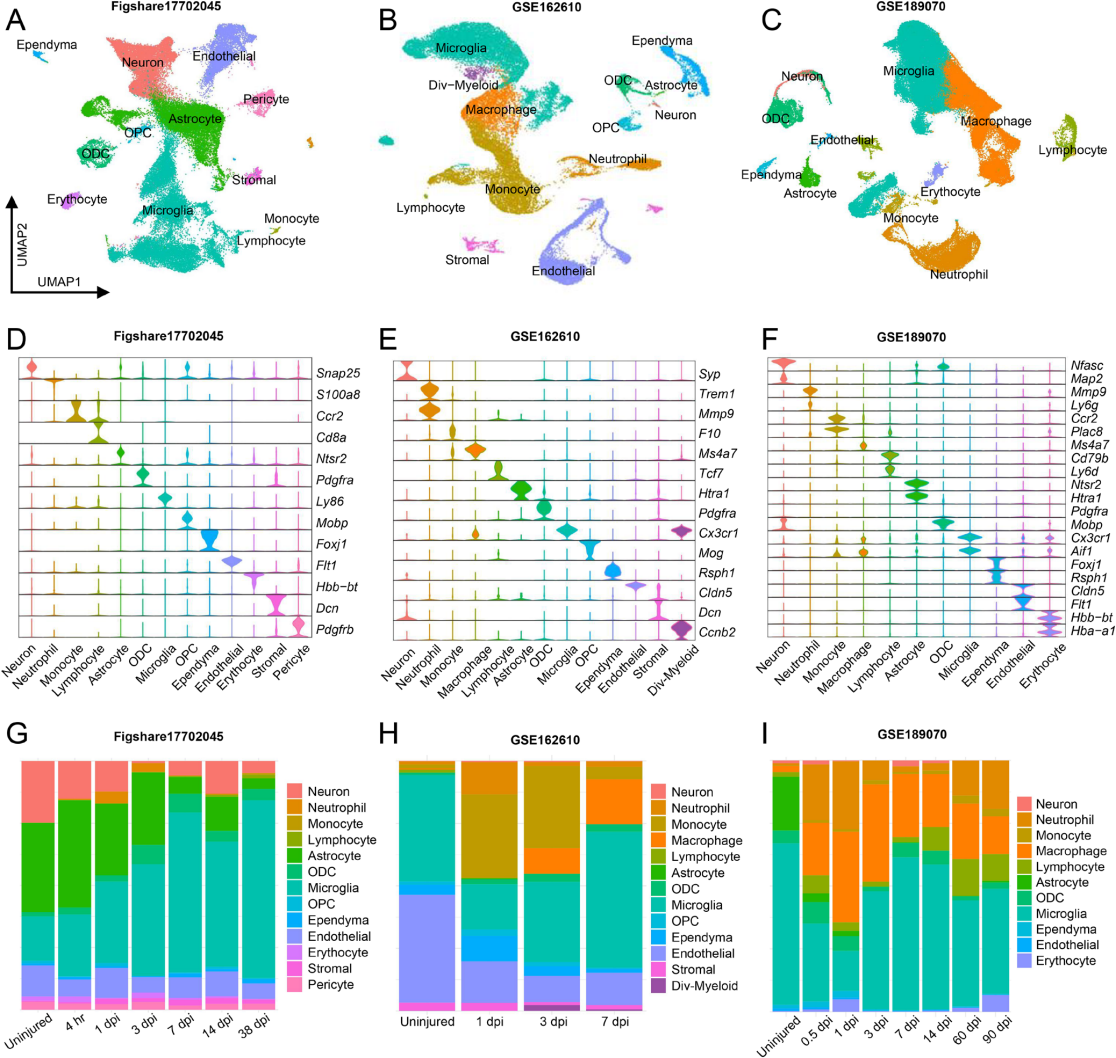
Single‐cell RNA sequencing (scRNA‐seq) analysis of spinal cord injury (SCI) in three datasets (figshare17702045, GSE162610, and GSE189070). (A–C) UMAP visualization plot of spinal cord cells sequencing in three databases. (D–F) Violin plots showing the signature genes of cells. (G–I) Relative frequency of different cell types in each experimental group.

UMAP visualization of three scRNA‐seq datasets of uninjured and injured spinal cord in different time points were conducted, with unsupervised clustering revealing major cell groups based on the expression of canonical genes (Figure 1A–C). Violin plots showed phenotypic markers for each group across the three datasets (Figure 1D–F). Comparative analysis revealed distinct alterations in cell proportions following injury (Figure 1G–I). Neurons and astrocytes decreased, while Microglia/macrophages began accumulating at 3 dpi and reach peak levels at 7 dpi. Neutrophils and lymphocytes were scarce in the uninjured spinal cord but appeared several hours after SCI.

**FIGURE 2:**
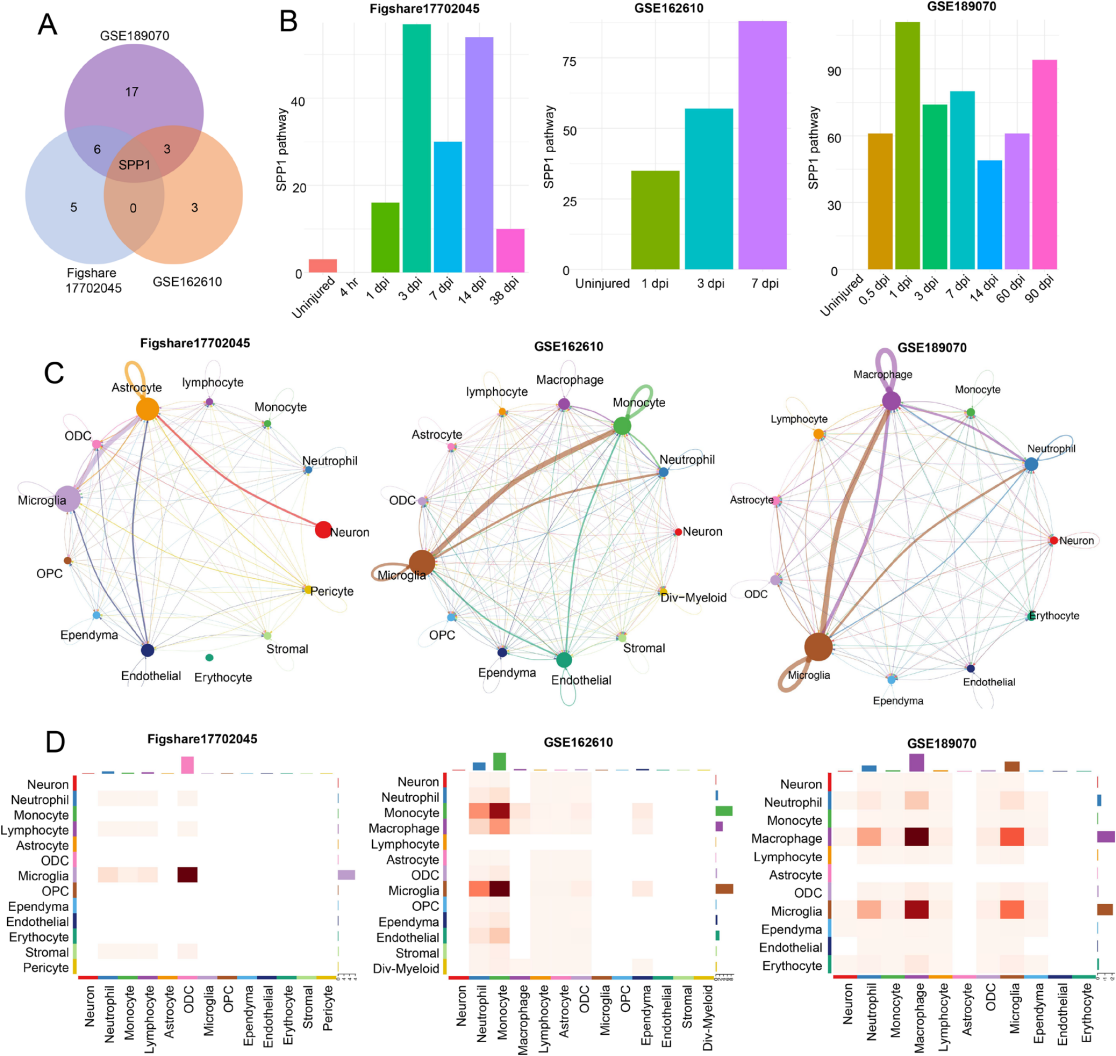
Cellchat analysis of secreted phosphoprotein 1 (SPP1) signaling pathway in three spinal cord injury (SCI) datasets. (A) Venn diagram comparing upregulated signaling pathways after SCI for each dataset. (B) Bar chart showing the number of SPP1 signaling network at different time points. (C) The number of SPP1 signaling network between different cell type. (D) The strength of SPP1 signaling network between different cell type.

To investigate the differences in intercellular communication between the SCI and sham groups, we analyzed receptor and ligand pathway expression in the cells. Single‐sample *t*‐test and single‐sample Wilcoxon signed‐rank test were performed to compare pathways between the sham and SCI groups (*p* < 0.05), and the identified upregulated pathways were intersected (Figure 2A and Table S1). Notably, SPP1 was the only signaling pathway co‐upregulated across all three datasets. The SPP1 signaling pathway was barely enriched in the uninjured group but was upregulated immediately after injury and persisted for several days (Figure 2B). Further analysis indicated that SPP1 signaling pathway was highly enriched in microglia/macrophages and others (monocytes, astrocytes, etc.) (Figure 2C,D).

### 3.2 SPP1 Is Elevated After SCI

**FIGURE 3:**
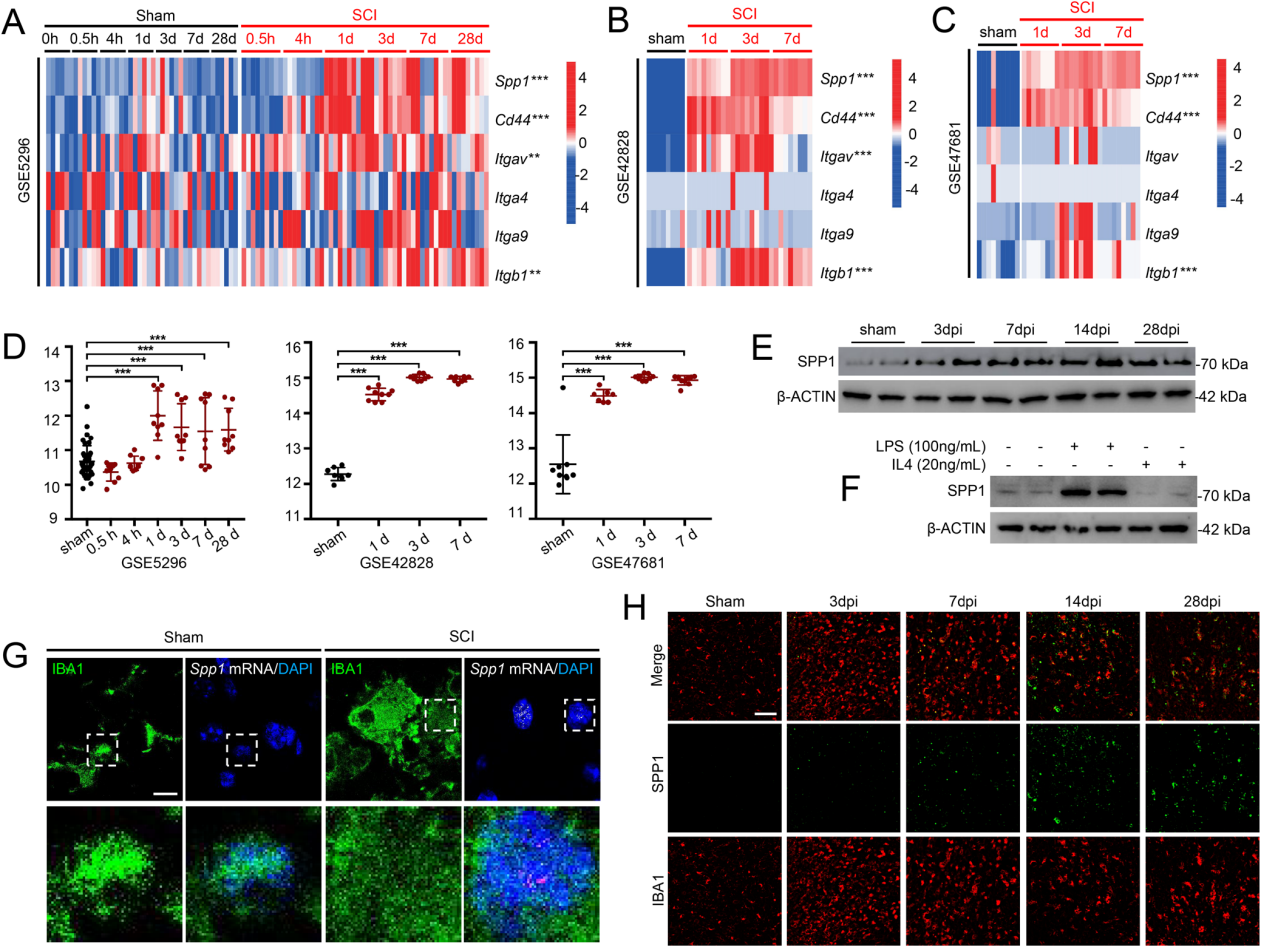
*Spp1* expression is upregulated after spinal cord injury (SCI). (A–C) Expression heatmap of ligands and receptors of SPP1 pathway in GSE5296, GSE42828, and GSE47681; **p, 0.01, ***p, 0.001. (D) Expression of *Spp1* in GSE5296, GSE42828, and GSE47681; ***p, 0.001. (E) Relative SPP1 protein level of spinal cord at designated time points post‐SCI. (F) Relative SPP1 protein level of spinal cord stimulated by LPS or IL‐4. (G) RNAscope in situ hybridization combined with immunofluorescence staining showing that *Spp1* was enhanced after SCI and localized to microglia (IBA1). Scale bar = 10 μm. (H) Immunofluorescence analysis revealing the colocalization of SPP1 protein and microglia (IBA1) at different time points. Scale bar = 50 μm. Student's two‐tailed unpaired *t*‐test (A–C). One‐way ANOVA followed by post hoc Bonferroni correction (D). SPP1, secreted phosphoprotein 1.

Differential expression analyses of all genes in the SPP1 signaling pathway (*Spp1*, *Cd44*, *Itgav*, *Itga4*, *Itga9*, and *Itgb1*) were conducted to explore their roles in the injured spinal cord. The expression of *Spp1*, *Cd44*, *Itgav*, and *Itgb1* were upregulated in SCI groups, compared with sham‐operated groups (Figure 3A–C). Additionally, *Spp1* was significantly upregulated after SCI in GSE5296 (Days 1, 3, 7, and 28 postinjury), GSE42828 (Days 1, 3, and 7 postinjury), and GSE47681 (Days 1, 3, and 7 postinjury) (Figure 3D). Increased SPP1 protein levels were observed after SCI, peaking at Day 14 post‐injury (Figure 3E). To emulate “M1” and “M2”‐like phenotypes in microglia, we treated primary microglia cultures with LPS (100 ng/mL) and IL‐4 (20 ng/mL) for 4 h. SPP1 level was upregulated in response to LPS stimulation, suggesting the potential role in mediating inflammation (Figure 3F). RNAscope in situ hybridization combined with immunofluorescence staining exhibited that *Spp1* was mainly located in surviving microglia (Figure 3G), which was consistent with previous study [28]. Besides, the time‐dependent immunofluorescence staining showed that SPP1 was secreted from microglia to extracellular matrix in the late stage of SCI (> 7 days), potentially contributing to increased intercellular communication between microglia and other cell types (Figure 3H).

### 3.3 SPP1 Enhances Neuron Maturation and Axon Mitochondrial Energetics

**FIGURE 4:**
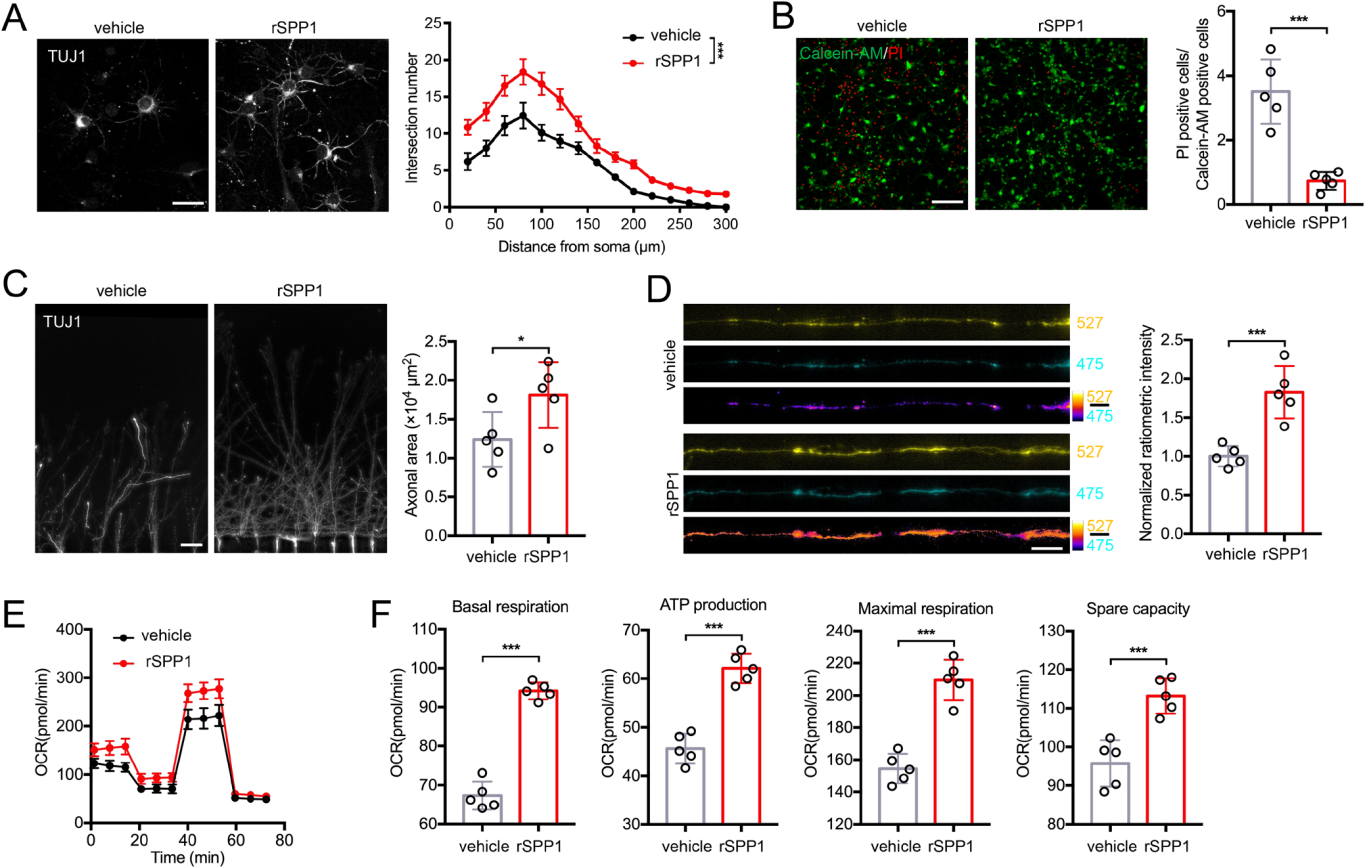
Secreted phosphoprotein 1 (SPP1) enhances the growth, survival, and high‐energy metabolism of neurons. (A) Representative images and sholl analysis of primary neurons treated with or without rSPP1. Scale bar = 10 μm. (B) Viability analysis of neurons treated with or without rSPP1 was analyzed by Calcein‐AM/PI double staining. Scale bar = 100 μm. (C) Immunofluorescence staining of axonal chambers, and quantification of regenerated axonal area. Scale bar = 100 μm. (D) Image and quantitative analysis of ATP levels in axons treated with or without rSPP1. Scale bar = 5 μm. (E, F) Quantification of OCR and measurement of basal respiration, ATP production, maximal respiration and spare capacity based on Seahorse extracellular flux analysis. Two‐way ANOVA followed by post hoc Bonferroni correction (A); Student's two‐tailed unpaired *t*‐test (B, C, D, F).

Primary neurons were cultured in vitro to model the effect of secreted SPP1 on neurons. Sholl analysis showed neurons treated with rSPP1 exhibited longer axon, more branches, and wider axon area compared to the vehicle group (Figure 4A). Neurons were subsequently subjected to OGD/R treatment, and compared to the vehicle group. Calcein‐AM/PI double staining demonstrated that rSPP1 increased neuronal viability (Figure 4B). Neurons were cultured in the somatodendritic chamber of microfluidic devices supplemented with rSPP1 to model the influence on axonal growth after binding to neuronal surface receptors. Axons were restricted to grow into the axonal chamber, and the rSPP1 group exhibited enhanced axonal growth (Figure 4C). Then neurons were infected with fluorescence resonance energy transfer (FRET)‐based ATP sensor GO‐A Team to investigate whether SPP1 affects neuronal mitochondrial function. It could emit at 527 nm (YFP) when bound to ATP and 475 nm (CFP) when free from ATP. Higher 527 nm/475 nm ratios indicate increased cytosolic ATP levels, and axons treated with rSPP1 exhibited greater mitochondrial energetics compared to the vehicle group (Figure 4D). Besides, Seahorse extracellular flux analysis was applied for neuronal energetics measurement (Figure 4E). After 24 h of rSPP1 treatment, neurons exhibited a significant increase in basal respiration, ATP production, maximal respiration, and spare capacity (Figure 4F).

### 3.4 SPP1 Aids Functional Recovery and Restricts Infiltration of Inflammatory Cells After SCI

**FIGURE 5:**
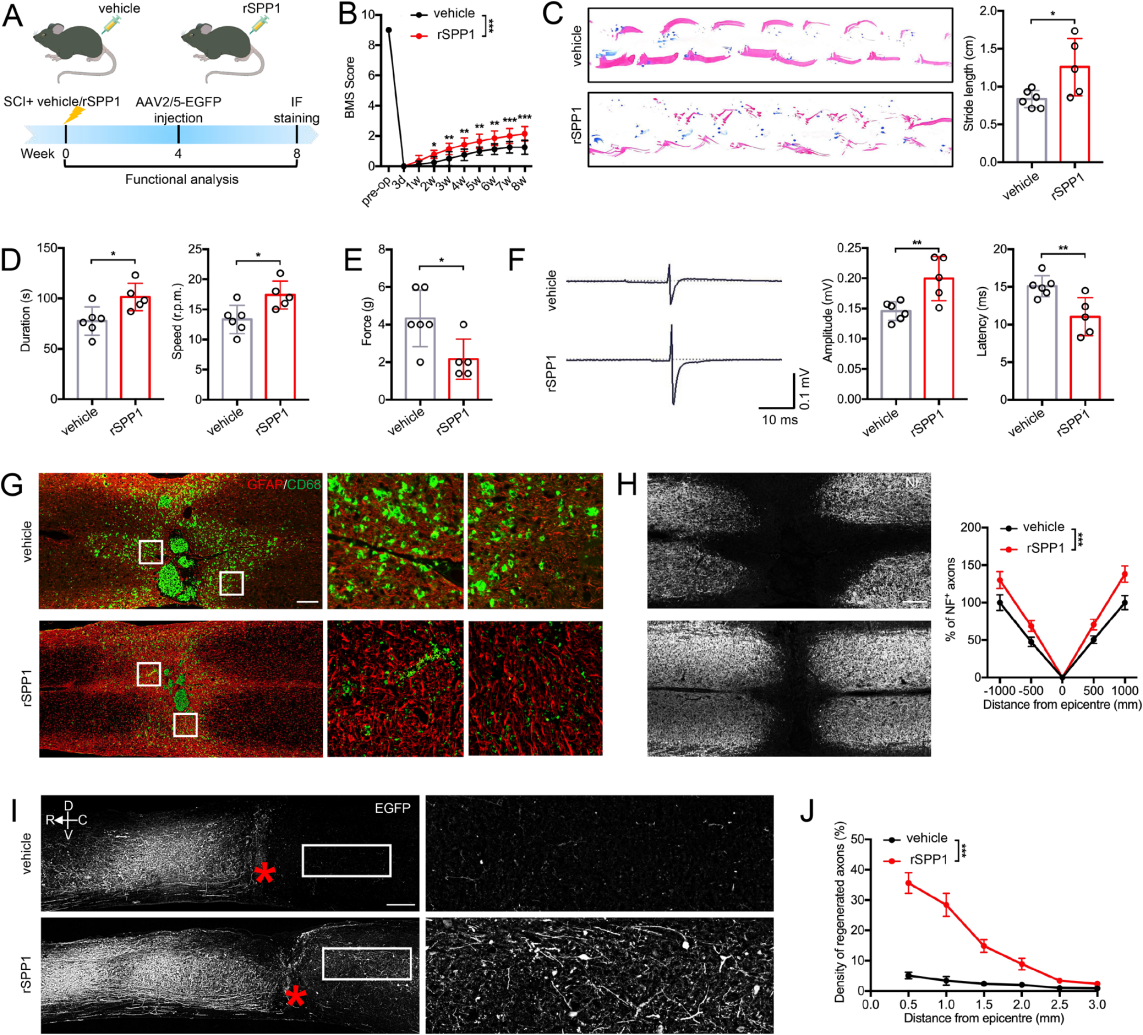
Secreted phosphoprotein 1 (SPP1) promotes functional recovery after spinal cord injury (SCI). (A) Schematic diagram illustrating the experimental design. (B) BMS scores during 28 days after SCI showed better recovery in rSPP1‐treated mice compared with vehicle‐treated mice. (C) Footprints representing animal gait at 28 days post‐SCI and quantification of footprint analysis outcomes for individual mice. Blue: Frontpaw print; red: Hindpaw print. (D) Rotarod tests of rSPP1‐ and vehicle‐treated mice at Day 28 postinjury. (E) Sensory behaviors by von Frey test of rSPP1‐ and vehicle‐treated mice at Day 28 postinjury. (F) Representative MEP amplitude and latencies in rSPP1‐ and vehicle‐treated mice at Day 28 post‐injury. (G) Representative immunofluorescence images for GFAP (astrocytes), and CD68 (macrophage/microglia) in rSPP1‐ and vehicle‐treated mice at Day 28 post‐injury. Scale bar = 200 μm. (H) Quantification of NF^+^ axons at various distances from lesion center of rSPP1‐ and vehicle‐treated mice at Day 28 post‐injury. Scale bar = 200 μm. (I) Representative immunofluorescence images of axons labeled with AAV2/5‐EGFP at Day 28 post‐injury. Scale bar = 200 μm. (J) Quantification of regenerated axon density at various distances from lesion center at Day 28 post‐injury. Two‐way ANOVA followed by post hoc Bonferroni correction (B, H, J); Student's two‐tailed unpaired *t*‐test (C, D, E, F).

To evaluate the role of SPP1 in SCI, we administered rSPP1 protein into the injured spinal cord immediately after the injury. Then functional behavioral analyses were performed on rSPP1‐ and vehicle‐treated mice at specified time points post‐injury (Figure 5A). Both the BMS score and footprint analysis demonstrated that administration of rSPP1 enhanced functional recovery (Figure 5B,C). Besides, rotarod testing indicated that rSPP1‐treated mice exhibited better motor coordination ability, including prolonged rotarod duration and improved motor coordination (Figure 5D). In addition to motor ability, rSPP1 injection could increase mice tactile sensitivity at Day 28 postinjury (Figure 5E). rSPP1 significantly reduced latencies and increased the amplitude of mice MEPs, which showed remodeling effect on hindlimb nerve conduction function after SCI (Figure 5F). After spinal cord injury, astrocytes adjacent to the lesion exhibited hypertrophy and elongation, consistent with reactive astrogliosis. Notably, these astrocytes relocate to the lesion center, progressively preventing inflammatory cell infiltration. Less infiltration of inflammatory microglia (CD68^+^) and decreased lesion areas were observed in rSPP1‐treated mice (Figure 5G). The NF is regarded as an essential element in neural network and axonal transport. We observed that rSPP1 significantly enhanced NF1 axon regeneration after SCI (Figure 5H). Functional restoration is widely believed to rely on axonal regeneration across the lesion site. Mice treated with rSPP1 exhibited a significant higher number of regenerated axons crossing the lesion site (Figure 5I). Furthermore, the density of regenerated axons in rSPP1‐treated mice was higher than in the vehicle group at various distances from the lesion border (Figure 5J).

### 3.5 *Spp1* Is Involved in the Activation and Proliferation of Microglia

After extracting microglia, subgroup analysis led to their re‐clustering into “homeostatic”, “activated”, “interferon (IFN)‐responsive”, and “proliferating” categories (Figure S1A), as described previously [29]. After injury, microglia underwent a notable transformation, shifting from expressing homeostatic genes to upregulating markers of activation, proliferation (*Mki67*), and IFN responsiveness (*Ifitm3*, *Isg15*, *Irf7*, and *Stat1*) (Figure S1B). Besides, significantly higher *Spp1* expression was observed in the activated and proliferating microglia (Figure S1C). To assess the alterations in differentiation trajectories and the role of *Spp1* during microglia progression, we conducted pseudotemporal analysis on all microglia. Notably, homeostatic microglia were mainly present in the initial stages of the trajectory, evolving towards activated, proliferating, and IFN‐responsive microglia as time progressed (Figure S1D). After Day 7 post‐injury, proliferative microglia decreased, while homeostatic microglia re‐emerged (Figure S1E). Consistent with pseudotemporal dynamics, *Spp1* expression notably increased as the progression continued (Figure S1F).

### 3.6 *Spp1* Is Upregulated by H4K12la in Glycolysis Reprogrammed Microglia After SCI

**FIGURE 6:**
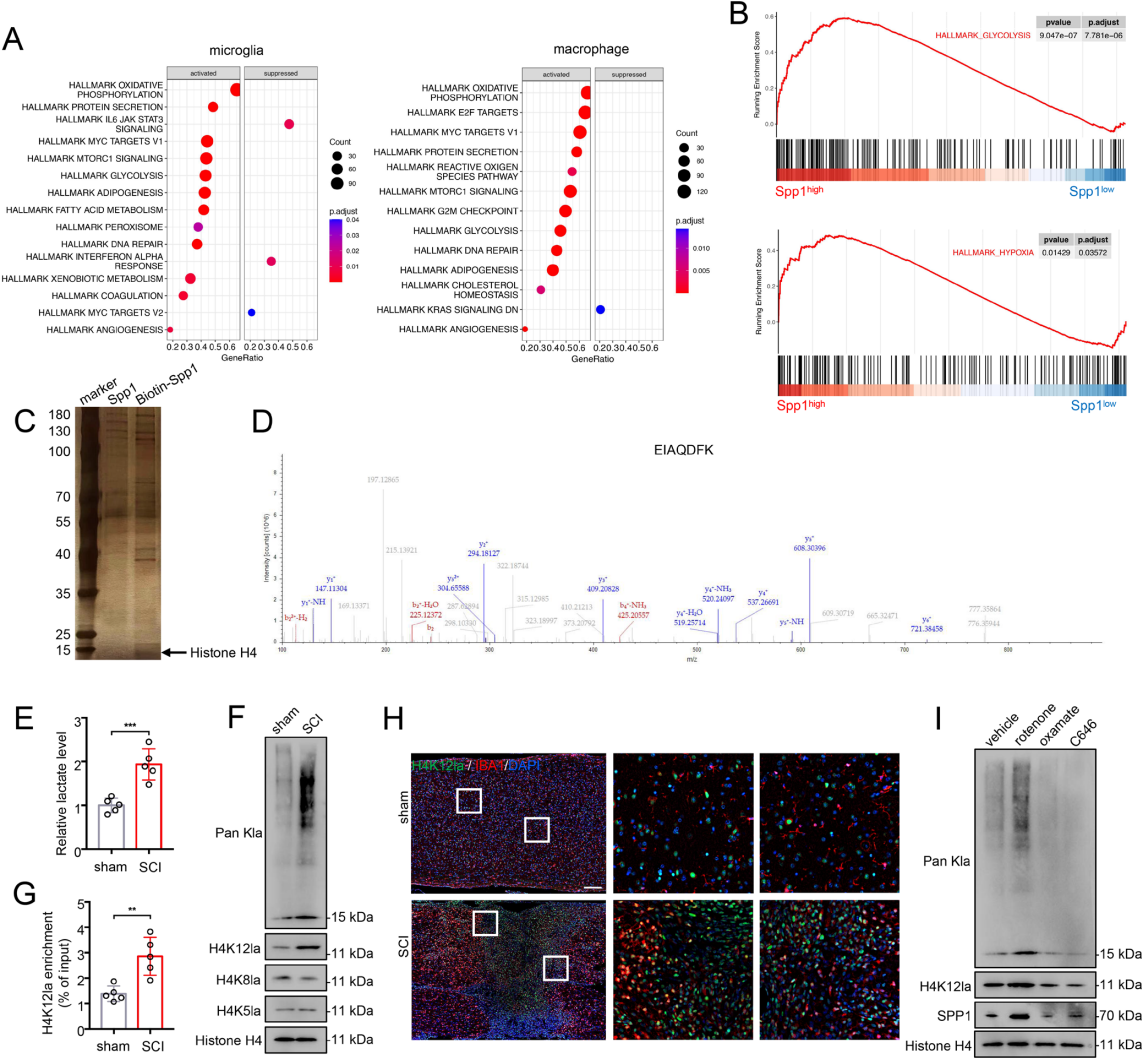
Histone H4 lysine 12 lactylation (H4K12la) in glycolytic reprogrammed microglia elevates *Spp1* expression after spinal cord injury (SCI). (A) Single‐gene GSEA analysis of *Spp1* in microglia and macrophages. (B) GSEA plot of glycolysis and hypoxia pathway in microglia. (C, D) DNA pull‐down assay showing proteins interacting with *Spp1* promoter. Bound proteins were analyzed with silver staining (C) and mass spectrometry (D). (E) Lactate levels in microglia from isolated SCI and sham mice. (F) Western blotting analysis of Pan‐ and site‐specific histone lactylation in microglia isolated from SCI and sham mice. (G) qChIP analysis of the *Spp1* promoter was performed using antibodies against H4K12la in microglia isolated from SCI and sham mice. (H) Immunofluorescence analysis showing the colocalization of H4K12la and microglia (IBA1) in SCI and sham mice. Scale bar = 200 μm. (I) Western blotting analysis of Pan Kla, H4K12la, and SPP1 in microglia under the treatment of rotenone, oxamate and C646. Student's two‐tailed unpaired *t*‐test (E, G).

To elucidate the underlying mechanism of *Spp1* upregulation after SCI, we conducted single‐gene GSEA analysis in microglia/macrophages based on scRNA‐seq data. Interestingly, *Spp1* exhibited significant associations with glycolysis and hypoxia pathways in both microglia and macrophages (Figure 6A,B). DNA‐pulldown assay also revealed that histone H4 bound more to the promoter region of *Spp1* in microglia (Figure 6C,D). Recent studies have elucidated the relationship between energy metabolism and histone modification [21, 30]. Certain byproducts of glycolytic reprogramming, such as lactate, act as epigenetic modulators. Therefore, we propose that microglia may undergo glycolytic reprogramming after SCI, resulting in the elevation of *Spp1* through histone lactylation. We first measured the lactate levels of microglia isolated from SCI and sham mice. The colorimetric assay indicated that lactate levels in microglia significantly increased after SCI (Figure 6E). Western blot analysis revealed elevated levels of both pan‐lysine lactylation (pan Kla) and H4K12la after SCI (Figure 6F). Additionally, qChIP analysis demonstrated that H4K12la levels on the *Spp1* promoter were significantly higher in microglia isolated from SCI mice compared to sham mice (Figure 6G). Immunofluorescence co‐staining revealed that H4K12la is specifically elevated after SCI and predominantly localized in microglia (Figure 6H). Lactate production is regulated by the balance between glycolysis and mitochondrial metabolism. Rotenone functions as an inhibitor of mitochondrial respiratory chain complex I, promoting cells to shift towards glycolysis. Oxamate, an inhibitor of lactate dehydrogenase (LDH), impedes lactate production, while C646, a histone acetyltransferase (HAT) inhibitor, reduces histone acetylation. As expected, microglia treated with rotenone exhibited increased levels of Pan Kla, H4K12la, and SPP1 compared to the vehicle group, while the opposite trend was observed in the oxamate and C646 groups (Figure 6I).

## 4 Discussion

In this study, we investigated the receptor‐ligand signaling pathways that were stably activated following SCI using scRNA‐seq, highlighting the SPP1 pathway as a central hub for intercellular communication. SPP1 positively regulates the activation and proliferation of microglia, facilitating functional and physiological recovery following SCI. We then confirmed the role of SPP1 in regulating neuronal maturation, axonal mitochondrial energetics, and inflammatory cell infiltration, which has not been previously reported. Finally, we demonstrated that H4K12la, induced by the reprogramming of glycolytic metabolism in microglia, promotes *Spp1* transcription.

Insights into cell communication indicated that the SPP1 signaling pathway is enriched in the interactions between microglia/macrophages and other cell types. SPP1 is a phosphorylated acidic glycoprotein adhesion molecule that is frequently upregulated during event‐induced inflammation, including infection [31, 32, 33], trauma [34, 35], allergy [36, 37, 38], and autoimmunity [39, 40, 41], performing biological functions through autocrine and/or paracrine signaling. Generally, SPP1 is recognized for its anti‐inflammatory properties in regulating inflammation and remodeling tissue [42]. Additionally, SPP1 has been shown to inhibit activation‐induced cell death caused by pathological events in macrophages, T lymphocytes, fibroblasts, and endothelial cells [43, 44]. Our research demonstrated that SPP1 participates in regulating immune and stromal cells through CD44 and integrin receptors in the lesion after SCI, consistent with prior studies [45]. CD44, a known receptor for SPP1, is a widely expressed multifunctional transmembrane glycoprotein primarily involved in the activation of immune and epithelial cells. Activation of CD44 has been shown to play a role in the initiation and progression of inflammation, with the CD44‐SPP1 interaction promoting cell migration from the bloodstream to the site of inflammation. Engagement of CD44 and SPP1 can disrupt the trimerization of Fas, thereby preventing the activation of the apoptotic pathway and enhancing resistance to cell death [46]. Furthermore, in coordination with CD44 receptors, SPP1 influences cytoskeleton‐related functions, including cell motility, fusion, and survival [47, 48]. Integrins serve as receptors for SPP1 and can be upregulated following brain injury to facilitate inflammatory infiltration and tissue repair, while also promoting neurite growth [49]. In our study, four integrins were implicated in the SPP1 signaling pathway, with expressions of *Itgav* and *Itgb1* significantly elevated following SCI. Indeed, SPP1 activates signal transduction pathways via integrins and CD44, orchestrating both pro‐inflammatory and anti‐inflammatory responses, promoting tissue remodeling and functional repair, and providing protection against potentially fatal injuries.

Recent scRNA‐seq studies have shown that *Spp1* is upregulated in various models of CNS disease [50]. Under physiological conditions, *Spp1* exhibits low expression levels in the CNS. However, *Spp1* is notably upregulated in pathological conditions, including neurodegenerative diseases such as Alzheimer's disease, Parkinson's disease, and multiple sclerosis [51, 52, 53], as well as acute brain injuries like stroke and hypoxic–ischemic injury [53, 54, 55]. Interestingly, plasma SPP1 closely parallels brain‐derived SPP1 and has been recognized as a novel marker for acute brain injuries [45]. Most studies to date have consistently demonstrated the neuroprotective effects of SPP1 on neurological disorders. The neuroprotective mechanism of SPP1 in the CNS appears to be multifaceted. SPP1 demonstrates a dual role in neuroinflammation, exerting a potent chemotactic influence on macrophages, dendritic cells, and T cells [46, 56]. Furthermore, the breakdown of the vascular network is a critical factor contributing to secondary injury in the CNS. Unfortunately, the emerging vascular system often fails to deliver adequate blood flow, which is essential for promoting functional recovery [57, 58]. SPP1 promotes angiogenesis through the VEGF and AKT pathways and is recognized as a significant proangiogenic factor. In animal models of stroke and spinal cord injury, *Spp1* has been shown to promote angiogenesis and improve nerve function [14, 59]. Escape from programmed cell death (PCD) is another mechanism underlying the neuroprotective effects of SPP1. In addition to blocking Fas‐mediated apoptosis through the aforementioned mechanism involving CD44, SPP1 is also involved in the regulation of ferroptosis in Alzheimer's disease [60]. Furthermore, increasing evidence supports the role of mitochondrial dysfunction and energy deficiencies in the progression of neurodegenerative disorders [61, 62, 63]. Neurons must maintain a local ATP supply to distal axons and synapses to facilitate action potential generation, uphold ion gradients, and transmit neural signals [64, 65]. For the first time, we illustrate in this study that SPP1 can increase axonal ATP levels. Consistent with its high‐energy metabolism, increased basal respiration was observed in primary neurons. Based on these findings, we evaluated the behavior of mice injected with rSPP1, confirming *Spp1* as a potential target for SCI intervention.

As the resident immune cells of the CNS, microglia rapidly activate and become the primary sources of various inflammatory mediators in response to even minor perturbations in environmental homeostasis [66, 67]. Traditionally, microglia have been categorized into pro‐inflammatory M1‐like and anti‐inflammatory M2‐like phenotypes; however, recent advancements in scRNA‐seq reveal the oversimplification of this binary classification [68, 69]. Specifically, secondary clustering of microglia using scRNA‐seq reveals the high complexity and heterogeneity of these cells. Current understanding recognizes that microglia are remarkably versatile, capable of exhibiting a spectrum of activation states in response to stimuli, each associated with unique transcriptional profiles and functional responsibilities [70, 71]. Research has demonstrated that *Spp1* is involved in activating anti‐inflammatory microglia following subarachnoid hemorrhage, leading to reduced inflammation. This intriguing finding prompts speculation about a similar microglial transformation mechanism in SCI. Depletion of microglia may exacerbate tissue damage and impair functional recovery from SCI [72]. Our pseudotime analysis indicated that microglia quickly switch from a homeostatic state to activation and proliferation upon experiencing SCI. *Spp1* mitigates inflammatory responses after SCI by stimulating active and proliferative microglial states, potentially contributing to functional recovery. Microglia are thought to upregulate core transcriptional program characteristics in response to stress signals, recognized as disease‐associated microglia (DAM) [73, 74]. After the cessation of stress, DAM is passively removed to facilitate the restoration of the remaining homeostatic microglia population. Additionally, Lan et al. confirmed that *Spp1* is the most significantly upregulated gene in DAM isolated from various CNS disease models and identified it as a marker gene for DAM [50]. In summary, *Spp1* ^+^ microglia represent a distinct type of reactive microglia similar to DAM, which will guide our future research directions.

However, the mechanism by which microglial activation influences *Spp1* remains unclear. It has been shown that microglia could perform “Warburg” effect similar to tumor cells during stress conditions, utilizing glycolytic and fatty acid oxidation to fulfill energy requirements while suppressing OXPHOS [75, 76]. Following SCI, microglial activation is accompanied by glucose reprogramming to maintain surveillance and phagocytosis [16]. Recent studies have suggested intricate crosstalk between energy metabolism and histone modification, with metabolites from the glycolysis pathway and the tricarboxylic acid cycle (such as lactic acid, α‐ketoglutaric acid, acetyl‐CoA, etc.) serving as substrates for histone modification [77, 78]. The induction of histone lactylation following microglia glycolytic reprogramming has recently been demonstrated in Alzheimer's disease [21]. In light of this, we propose the hypothesis that glycolytic reprogramming‐induced lactate accumulation in microglia drives alterations in *Spp1* transcription through histone lactylation modifications. Our findings reveal that H4K12la specifically activates *Spp1* transcription in microglia, significantly contributing to SCI recovery. This discovery provides a pioneering analysis and valuable insights into metabolic reprogramming and epigenetic regulation, suggesting a novel approach to addressing SCI. Selectively promoting H4K12la, without affecting the energy metabolism of microglia, may represent a novel therapeutic approach for SCI treatment.

Despite the promising findings, this research has inherent limitations. First, *Spp1* is predominantly expressed in microglia, macrophages, and astrocytes, but this investigation primarily focuses on its role in microglia and macrophages. Additional studies are needed to explore the mechanisms by which SPP1 enhances reactive astrogliosis and substantial inflammatory infiltration. Second, loss‐of‐function approaches, such as small interfering RNA models, are necessary to demonstrate the potential functional role of *Spp1* in SCI. Finally, we were unable to generate a site mutation of histone H4 to assess its specific effect on *Spp1* transcription. In future studies, we will identify the lactylases and delactylases involved in writing, erasing, and reading H4K12la, providing further insights into the H4K12la/*Spp1* axis.

## 5 Conclusions

In summary, the activation of microglia following spinal cord injury (SCI) is associated with enhanced glycolytic reprogramming, wherein elevated levels of H4K12la drive *Spp1* transcription. SPP1 actively participates in intercellular communication and plays a crucial role in regulating neuronal maturation, axonal mitochondrial energetics, and inflammatory cell infiltration, ultimately contributing to improved functional recovery.

## Author Contributions

Xiaokun Wang, Geliang Zhou, and Junjun Xiong contributed equally to this work. The article is mainly conceived and written by Xiaokun Wang and Geliang Zhou. Wu Ye, and Yu Gao performed the literature search. Haofan Wang, and Dishui Pan designed the figure and table. Junjun Xiong and Wu Ye contributed to manuscript revisions. Zheng Zhou and Yongjun Luo designed and supervised this work. All authors have read and approved the final submission.

## Ethics Statement

The animal protocols were approved by the Animal Committee of the First Affiliated Hospital of Nanjing Medical University.

## Conflicts of Interest

The authors declare no conflicts of interest.

## Supporting information


**Figure S1.** *Spp1* participates in the progression of microglia. (A) UMAP visualization plot of microglia subclustered from figshare17702045. (B) Dot plots showing the expression of phenotyping markers for each population. Dot size indicates the percentage of cells in which that gene is detected, while the color bar corresponds to the average expression. (C) UMAP visualization plot of expression pattern of *Spp1*. Color bar indicates the average expression in each cell. (D) Pseudotime analysis showing the potential evolutionary trajectory of SCI microglia. (E) Relative frequency of different microglia subtype in each experimental group. (F) Expression alterations of *Spp1* in the pseudotime.

**Table S1.** The activation of signaling pathways in the uninjured group and SCI groups in three datasets.

Data S1.

## Data Availability

The data that support the findings of this study are available on request from the corresponding author.
