# Supplementary figures and images for: H4K12 Lactylation Activated‐Spp1 in Reprogrammed Microglia Improves Functional Recovery After Spinal Cord Injury
Source: CNS Neurosci Ther. 2025 Feb 12;31(2):e70232. doi: 10.1111/cns.70232 (PMC11821456; doi:10.1111/cns.70232)

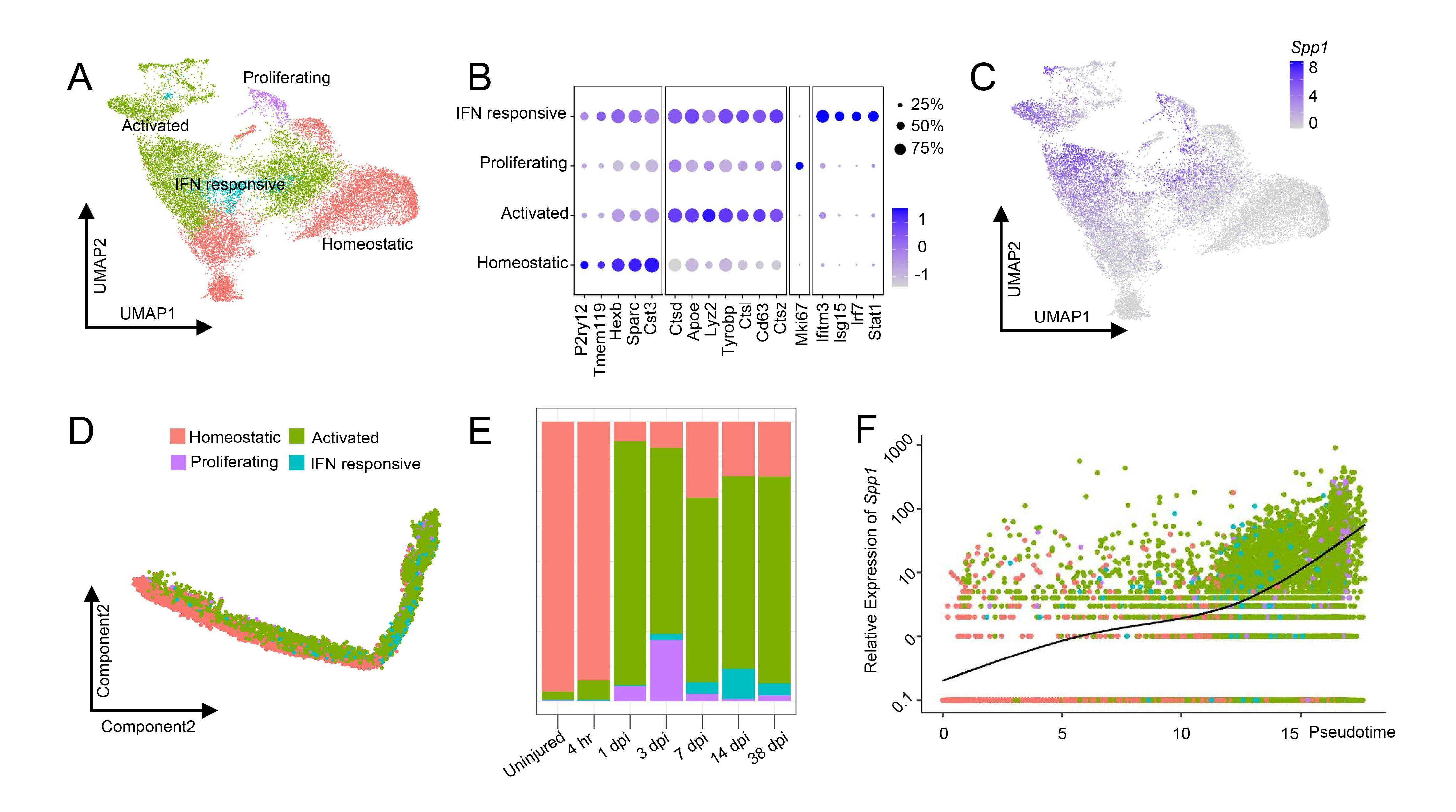

Supplement: Supplementary file 1 — Figure S1. Spp1 participates in the progression of microglia. (A) UMAP visualization plot of microglia subclustered from figshare17702045. (B) Dot plots showing the expression of phenotyping markers for each population. Dot size indicates the percentage of cells in which that gene is detected, while the color bar corresponds to the average expression. (C) UMAP visualization plot of expression pattern of Spp1. Color bar indicates the average expression in each cell. (D) Pseudotime analysis showing the potential evolutionary trajectory of SCI microglia. (E) Relative frequency of different microglia subtype in each experimental group. (F) Expression alterations of Spp1 in the pseudotime. [file CNS-31-e70232-s003.tif]
