# Supplementary material for: H4K12 Lactylation Activated‐Spp1 in Reprogrammed Microglia Improves Functional Recovery After Spinal Cord Injury
Source: CNS Neurosci Ther. 2025 Feb 12;31(2):e70232. doi: 10.1111/cns.70232 (PMC11821456; doi:10.1111/cns.70232)

Full unedited blot for Figure 3E

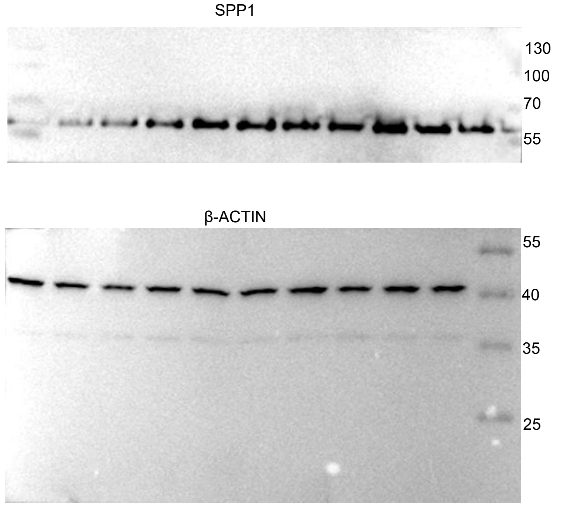

Full unedited blot for Figure 3F

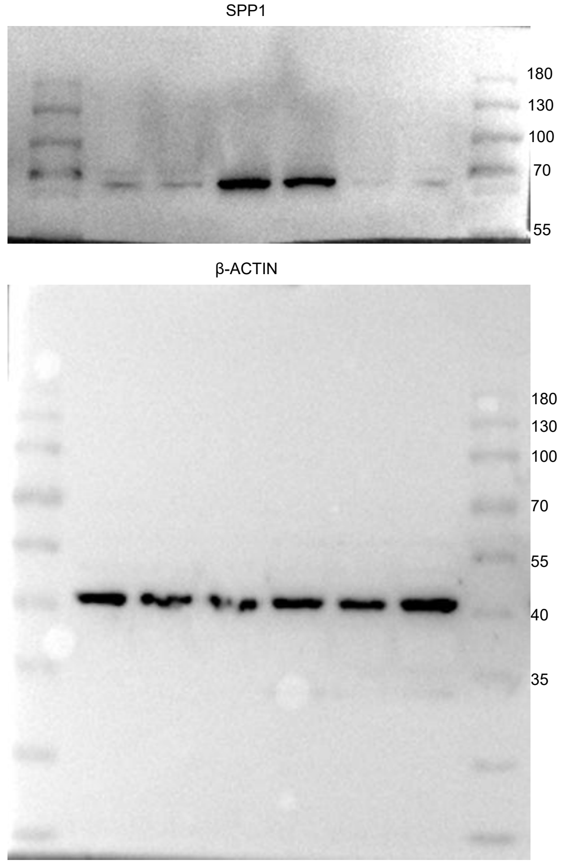

Full unedited blot for Figure 6F

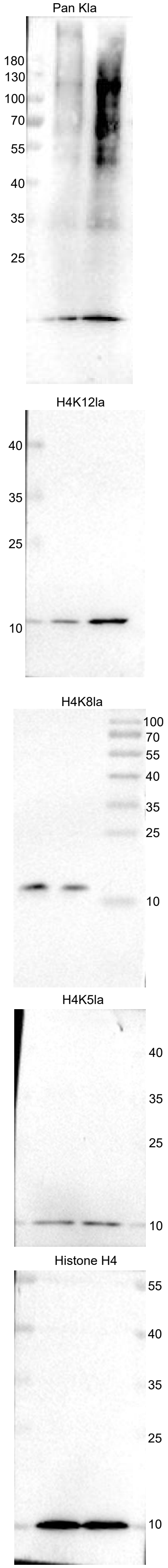

Full unedited blot for Figure 6I

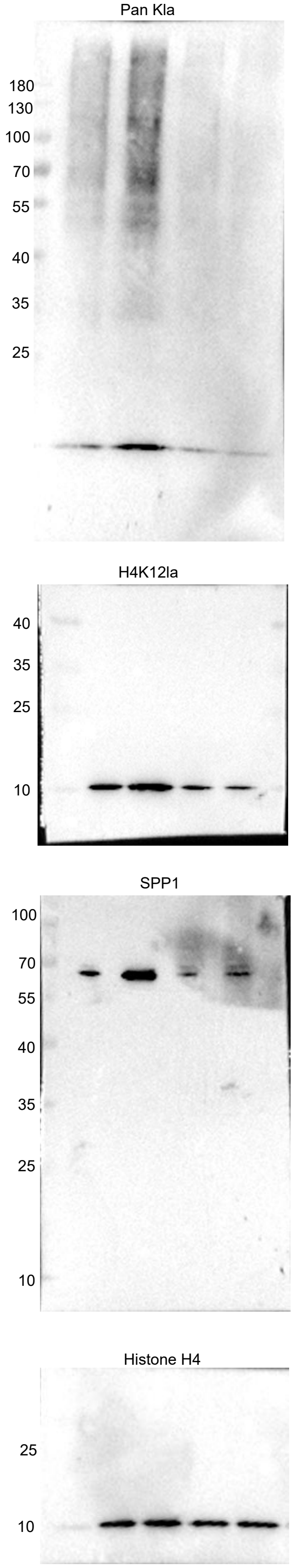

Supplement: Supplementary file 3 — Data S1. [file CNS-31-e70232-s002.pdf]
